# In Vitro Immunomodulatory Effects of Equine Adipose Tissue-Derived Mesenchymal Stem Cells Primed with a Cannabidiol-Rich Extract

**DOI:** 10.3390/ijms26094208

**Published:** 2025-04-29

**Authors:** Lorena Battistin, Luís Felipe Arantes Moya, Lucas Vinícius de Oliveira Ferreira, Aline Márcia Marques Braz, Márcio de Carvalho, Marjorie de Assis Golim, Rogério Martins Amorim

**Affiliations:** 1 Department of Veterinary Clinic, School of Veterinary Medicine and Animal Science, São Paulo State University (UNESP), Botucatu 18618-681, SP, Brazillv.ferreira@unesp.br (L.V.d.O.F.);; 2 Center for Translational Research in Regenerative Medicine, School of Veterinary Medicine and Animal Science, São Paulo State University (UNESP), Botucatu 18618-681, SP, Brazil; 3 Laboratory of Applied Biotechnology, Clinical Hospital of the Medical School, São Paulo State University (UNESP), Botucatu 18618-687, SP, Brazil

**Keywords:** cannabinoid receptors, cell therapy, cytokines, horse, phytocannabinoid

## Abstract

Cell-based therapy using mesenchymal stem cells (MSCs) shows promise for treating several diseases due to their anti-inflammatory and immunomodulatory properties. To enhance the therapeutic potential of MSCs, in vitro priming strategies have been explored. Cannabidiol (CBD), a non-psychoactive compound derived from cannabis, may influence MSC proliferation, differentiation, and immunomodulatory properties. This study evaluates the immunomodulatory potential of equine adipose tissue-derived MSCs (EqAT-MSCs) primed with a CBD-rich cannabis extract. EqAT-MSCs (P3) were primed with CBD concentrations of 5 µM and 7 µM for 24 h. Morphological analysis, MTT assay, β-galactosidase activity, apoptosis assays, and gene expression of interleukins IL-1β, IL-6, IL-10, interferon-gamma (IFN-γ), and tumor necrosis factor-alpha (TNF-α) were conducted. Additionally, cannabinoid receptor 1 (CB1) and 2 (CB2) expression were evaluated in naïve EqAT-MSCs (P2–P5). The naïve EqAT-MSCs expressed CB1 and CB2 receptors. Priming with 5 µM significantly increased the expression of IL-10, TNF-α, and IFN-γ, while 7 µM decreased IL-1β and IL-6 expression. No significant changes were observed in other cytokines, MTT, β-galactosidase activity, or apoptosis. These findings demonstrate that naïve EqAT-MSCs express CB1 and CB2 receptors and priming with the extract modulates the expression of pro- and anti-inflammatory cytokines, highlighting its potential immunomodulatory role in EqAT-MSC-based therapies.

## 1. Introduction

Cell-based therapy using mesenchymal stem cells (MSCs) represents a promising approach for the treatment of several diseases due to their anti-inflammatory, immunomodulatory, and anti-apoptotic properties [1,2].

The therapeutic effects of MSCs are primarily mediated through direct cell-to-cell interactions and the release of soluble factors and extracellular vesicles via paracrine mechanisms [3]. MSCs interact with several immune cells, including T lymphocytes, natural killer cells, neutrophils, and macrophages, thereby modulating the immune response [3,4].

MSCs can be derived from various tissues, including adipose tissue, bone marrow, and fetal tissues such as amniotic fluid, placenta, and umbilical cord [5]. In horses, adipose tissue is considered a reliable and easily accessible source of MSCs [6]. Importantly, studies in large animal models, including horses, not only address species-specific health and welfare needs, but also serve as a translational model for human diseases, given the shared pathophysiological similarities [7].

Despite the therapeutic potential of MSCs, post-transplantation, cell survival, and differentiation capacity of donor cells may be compromised due to adverse conditions of the microenvironment, which may lead to low engraftment rates, excessive cell death, and reduced therapeutic efficacy [8]. To optimize the therapeutic properties of MSCs, various priming strategies have been developed. Priming involves exposing MSCs to specific stimuli to enhance their biological functions, such as paracrine activity, regenerative potential, and immunomodulatory capacity [8,9].

For instance, MSC priming with pro-inflammatory cytokines, such as tumor necrosis factor-alpha (TNF-α) and interferon-gamma (IFN-γ), has been shown to enhance their immunoregulatory functions [10,11,12,13]. Additional strategies include genetic modification [14] and the use of optimized culture conditions [15,16].

In this context, Cannabidiol (CBD), a non-psychoactive phytocannabinoid derived from *Cannabis sativa*, has recently garnered attention for its anti-inflammatory and immunomodulatory properties in both human and veterinary medicine [17,18,19]. Based on these effects, CBD could be used as a priming agent to enhance the therapeutic potential of MSCs.

Previous studies have demonstrated that phytocannabinoids can modulate MSC proliferation and differentiation [20,21]. Among them, CBD has been shown to immunomodulate the expression of the genes associated with inflammation, immune response, and apoptosis in human MSCs [22,23]. In line with these findings, recent evidence shows that CBD reduces the TNF-α-induced upregulation of key pro-inflammatory cytokines in human dental pulp stem cells [24]. Similarly, tetrahydrocannabinol (THC) has been shown to enhance the immunomodulatory effects of mouse bone marrow-derived MSCs [25].

Furthermore, CBD potentiates the signaling of type 1 (CB1) and type 2 (CB2) cannabinoid receptors, key components of the endocannabinoid system [26]. This system plays a pivotal role in regulating a wide range of physiological functions, making it a potential target for the development of new treatments [27].

Thus, this study aims to evaluate the in vitro immunomodulatory potential of EqAT-MSCs following priming with a CBD-rich cannabis extract. The findings from this study may contribute to the development of more effective cell-based therapies for equine health and provide novel insights into the application of CBD as a priming agent. Additionally, these results may offer valuable data for translational approaches in regenerative medicine.

## 2. Results

### 2.1. Morphological Changes

**Figure 1:**
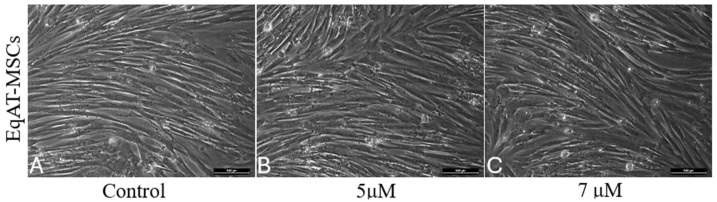
Morphological characterization of equine adipose tissue-derived mesenchymal stem cells (EqAT-MSCs). (**A**) Control EqAT-MSCs cultured in complete culture medium; (**B**) EqAT-MSCs primed with Cannabidiol (CBD)-rich cannabis extract at concentrations of 5 μM and (**C**) 7 μM. No morphological changes were observed compared to the control group. Magnification, ×200. Scale bar = 100 μM.

No differences in morphology were observed between the groups primed with CBD-rich cannabis extract and the control group after 24 h of priming (Figure 1).

### 2.2. Cellular Metabolic Activity

**Figure 2:**
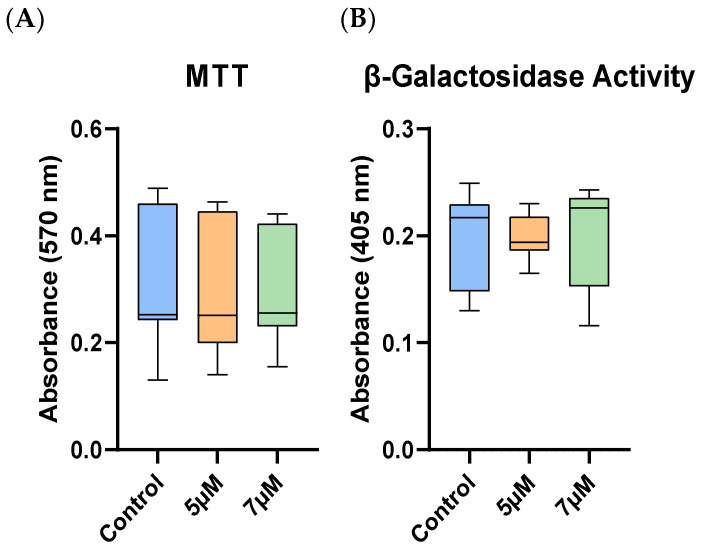
Cellular metabolic and β-galactosidase activities in equine adipose tissue-derived mesenchymal stem cells (EqAT-MSCs). No significant differences were observed after priming with cannabidiol (CBD)-rich cannabis extract in (**A**) cellular metabolic activity (MTT assay) and (**B**) senescence-associated β-galactosidase activity. Data are shown as medians, interquartile ranges, minimum, and maximum values. Kruskal–Wallis test.

Following the priming of the EqAT-MSCs with a CBD-rich cannabis extract, no significant differences in metabolic activity were observed among the experimental groups (Figure 2).

### 2.3. β-Galactosidase Activity

After priming with the CBD-rich cannabis extract, no significant differences in β-galactosidase activity were observed between the groups (Figure 2).

### 2.4. Apoptosis Detection

**Figure 3:**
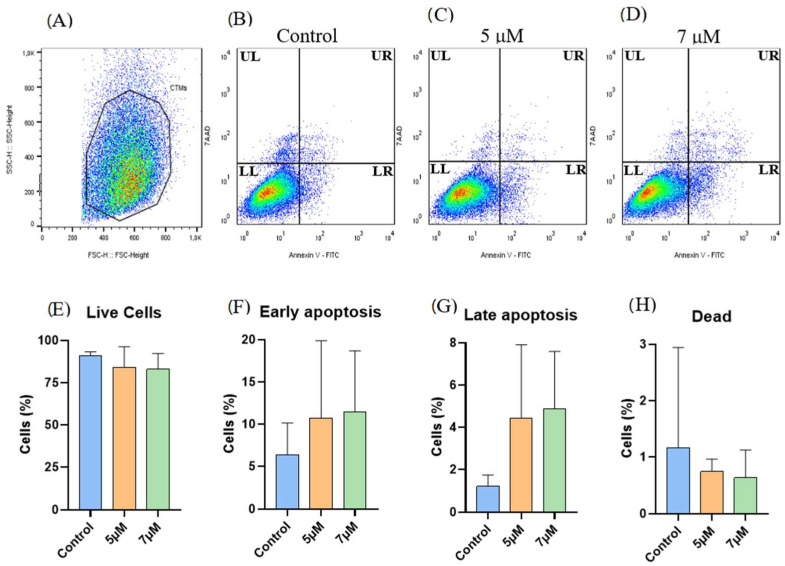
Apoptosis assay (Annexin V × 7-AAD): (**A**–**D**) Representative gating strategy for the analysis of cell viability using Annexin-V FITC and 7-AAD. (**A**) Selection of the cellular population. (**B**) Control group. (**C**) Equine adipose tissue-derived mesenchymal stem cells (EqAT-MSCs) primed with cannabidiol (CBD)-rich cannabis extract at concentrations of 5 μM (**D**) and 7 μM. Quadrants are labeled as follows: UL (Upper Left) represents dead cells, LL (Lower Left) represents live cells, UR (Upper Right) represents late apoptotic cells, and LR (Lower Right) represents early apoptotic cells. No significant changes were observed when comparing (**E**) the live cells, (**F**) early apoptotic cells, (**G**) late apoptotic cells, and (**H**) dead cells of the control group with EqAT-MSCs primed with CBD-rich cannabis extract at concentrations of 5 μM and 7 μM. Data are represented as the mean ± standard deviation, one-way analysis of variance (ANOVA).

Apoptosis assessment was performed on three samples of EqAT-MSCs, and no significant changes were observed when comparing the groups in this experiment (Figure 3).

### 2.5. Gene Expression of Cytokines and Cannabinoid Receptors

**Figure 4:**
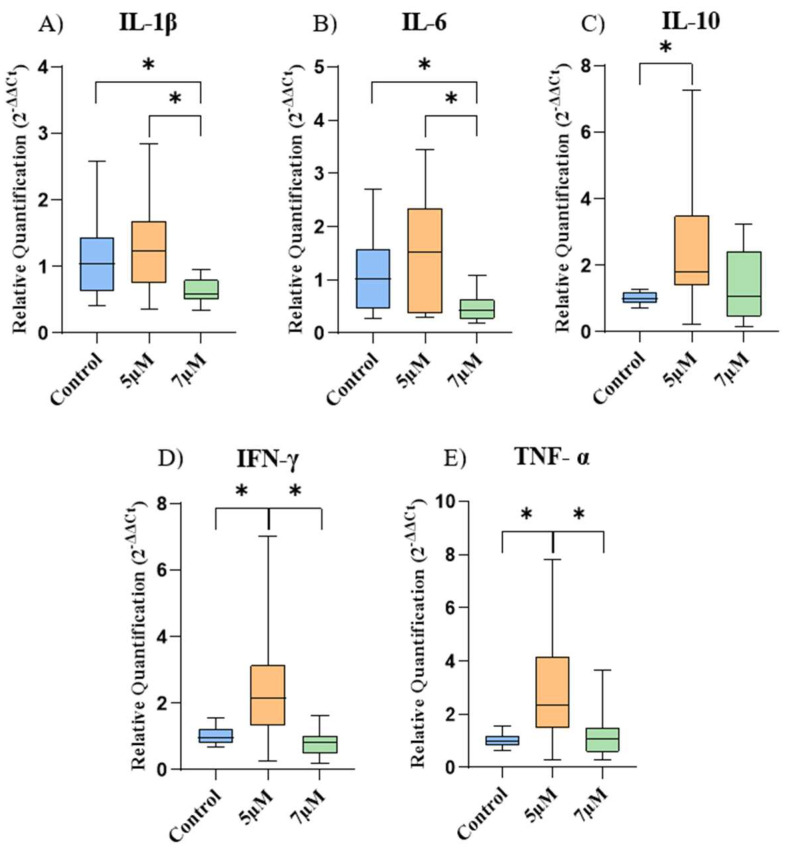
Relative expression of the cytokines among the experimental groups. (**A**) Interleukin 1 beta (IL-1β), (**B**) interleukin-6 (IL-6), (**C**) interleukin-10 (IL-10), (**D**) interferon gamma (IFN-γ), and (**E**) tumor necrosis factor-alpha (TNF-α). Data are represented as medians, interquartile ranges, and minimum and maximum values (* *p* < 0.05). Kruskal–Wallis and Dunn’s tests.

The gene expression of the cytokines is represented in Figure 4. In the EqAT-MSCs, there was a significant decrease in the gene expression of IL-1β and IL-6 after priming with a concentration of 7 μM compared to the control group. Regarding IL-10, IFN-γ, and TNF-α, a considerable increase was observed after priming with a concentration of 5 μM compared to the control group.

Furthermore, when comparing the cells among the different concentrations of CBD-rich cannabis extract used in this study, a significant increase in the expression of IL-1β, IL-6, IFN-γ, and TNF-α was noticed in the concentration of 5 μM compared to the concentration of 7 μM.

**Figure 5:**
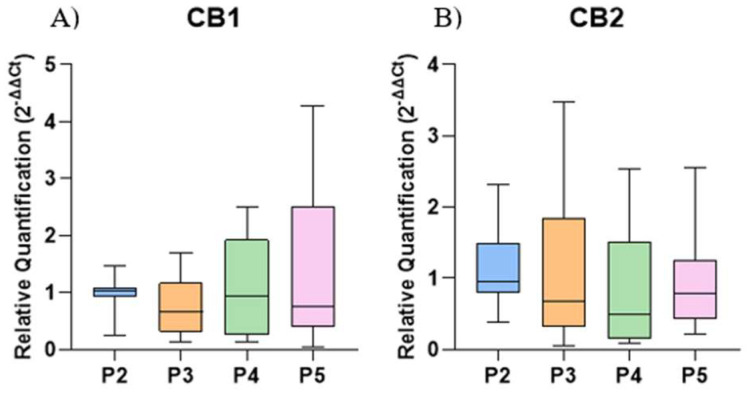
Relative expression of cannabinoid receptors 1 (CB1) and 2 (CB2) in the control group of naïve equine adipose tissue-derived mesenchymal stem cells (EqAT-MSCs) across different passages (P2–P5): (**A**) CB1. (**B**) CB2. No significant difference was observed between the passages. Data are represented as medians, interquartile ranges, minimum, and maximum values. Kruskal–Wallis test.

There was no significant difference in the gene expression of CB1 and CB2 among the different culture passages compared to P2 (Figure 5).

## 3. Discussion

MSCs possess significant therapeutic potential; however, challenges such as limited clinical efficacy, functional quiescence post-transplantation, poor engraftment, and cellular senescence continue to hinder their clinical application [28]. To address these limitations, various priming strategies have been developed to enhance MSC functionality and improve therapeutic outcomes. These include 3D culture, growth factor stimulation, hypoxic conditions, electrical and thermal stimulation, the use of chemical agents and drugs, and genetic modifications [8,29].

In this context, phytocannabinoids such as CBD have attracted attention due to their ability to interact with a wide range of molecular targets within the endocannabinoid system [22], exhibiting anti-inflammatory, antioxidant, and neuroprotective properties, rendering it a promising candidate for the treatment of several diseases [30]. Furthermore, studies have demonstrated that CBD can enhance the proliferation, self-renewal, and migration of MSCs [31,32].

Cannabinoid receptors CB1 and CB2 are well-characterized components of the endocannabinoid system [33]. These receptors have been previously identified in mouse embryonic stem cells [34], mouse BM-MSCs [25], and human AT-MSCs [35]. In this study, we demonstrate the gene expression of CB1 and CB2 in EqAT-MSCs, suggesting the conservation of these receptors across mammalian species [36]. This finding supports the potential for utilizing synthetic or natural agonists to modulate these receptors, thereby enhancing the anti-inflammatory and immunomodulatory functions of MSCs.

The concentrations of 5 and 7 µM of CBD-rich cannabis extract, as well as the priming duration, were selected based on previous studies [23,37]. Moreover, we demonstrated that neither concentration affected cell metabolic activity, viability, or β-galactosidase activity, indicating their safety.

A morphological analysis revealed no structural alterations following priming, consistent with previous findings in human MSCs [23], indicating that CBD concentrations of 5 μM and 7 μM preserved cellular integrity after 24 h.

The anti-apoptotic effects of CBD have been previously described in human gingival-derived MSCs [23] and human adipose-derived mesenchymal stem cells [22]. However, the apoptosis assays in this study revealed no significant differences in the EqAT-MSCs primed with 5 μM or 7 μM CBD compared to the control group, suggesting that these concentrations do not impact apoptosis rates in the EqAT-MSCs. In addition, no alterations were observed in cell metabolic or β-galactosidase activity. Collectively, these findings indicate that CBD priming does not induce cytotoxic effects, suggesting it may be safely employed in future studies.

CBD is well known for its anti-inflammatory properties. So, we evaluated the expression of several cytokines after the priming of the EqAT-MSCs. Although a study reported an increase in the gene expression of IL-1β and IL-6 in human adipose-derived stem cells after priming with CBD at a concentration of 5 μM [22], we did not observe a significant difference using the same concentration. However, there was a significant decrease in IL-1β and IL-6 after priming at a concentration of 7 µM. This can be explained by the fact that CBD exerts complex effects on the regulation of inflammatory cytokine expression through multiple cellular mechanisms. This phytocannabinoid can inhibit the activation of the nuclear factor kappa-light-chain-enhancer of the activated B cell (NF-κB) pathway, a key regulator of inflammation, thereby reducing the expression of inflammatory cytokines [38,39]. Additionally, CBD can modulate the activity of the NOD-like receptor pyrin domain-containing 3 inflammasome (NLRP3 inflammasome), which is involved in the activation of caspase-1 and the subsequent release of inflammatory cytokines [23,40,41]. These findings suggest that priming with CBD-rich cannabis extract at a concentration of 7 μM could enhance the anti-inflamatory capacity of the EqAT-MSCs.

IL-10 is a key cytokine in immune regulation that plays an essential role in modulating inflammatory processes [42]. We observed a significant increase in IL-10 gene expression after the priming of the EqAT-MSCs with CBD at a concentration of 5 μM. CBD may enhance the activation of signal transducer and activator of transcription 3 (STAT3) and promote the increase in IL-10 synthesis [43]. In addition, another study reported that THC can increase IL-10 secretion, which could be attributed to CB2 receptor activation via the ERK 1/2 pathway [25]. However, our result contrasts with a previous study that used the same concentration to stimulate human adipose-derived mesenchymal stem cells and reported a decrease in the expression of this cytokine [22]. IL-10 can suppress antigen-presenting cells, inhibit T-cell proliferation, and promote the activity of regulatory T (Treg) cells [44,45]. The increase in IL-10 is of great interest in regenerative medicine due to its anti-inflammatory and regulatory properties, which are consistent with the known effects of CBD.

Despite previous reports demonstrating that CBD decreases TNF-α and IFN-γ in vitro in equine peripheral blood mononuclear cells [46], attenuates TNF-α-induced TNF-α expression in human dental pulp stem cells [24], and reduces TNF-α in vitro after the stimulation of human adipose-derived mesenchymal stem cells [22] and IFN-γ in senior horses [47]; we identified a significant increase in the expression of both cytokines after priming with CBD-rich cannabis extract at a concentration of 5 µM. These results deviate from the well-known anti-inflammatory mechanisms of CBD. Even though TNF-α and IFN-γ are often associated with harmful effects, they can be beneficial in specific contexts. TNF-α is a key cytokine in the inflammatory response, essential for defending against infections [48], and IFN-γ is crucial in cell-mediated immunity, coordinating various antimicrobial functions, inducing antiviral responses, and exhibiting antiproliferative effects on cancer cells [49].

This preliminary study is limited by the absence of cytokine protein analysis and the lack of assessment of the secretory activity of the equine EqAT-MSCs following priming with various concentrations of CBD-rich cannabis extract.

## 4. Materials and Methods

### 4.1. Experimental Design

The EqAT-MSCs were thawed and cultured in a complete medium containing 90% Dulbecco’s modified Eagle’s medium DMEM/F12, 10% fetal bovine serum (FBS) (both from Nova Biotecnologia, Cotia, SP, Brazil), 1% penicillin/streptomycin, and 0.5% amphotericin B (both from Gibco, Grand Island, NY, USA). The cells were primed with CBD-rich cannabis extract for 24 h.

**Figure 6:**
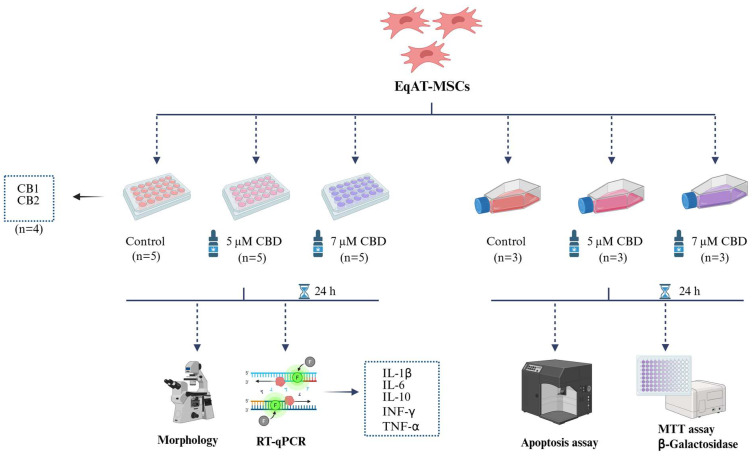
Experimental design. Equine adipose tissue-derived mesenchymal stem cells (EqAT-MSCs) (P3) were primed with 5 µM of cannabidiol (CBD)-rich cannabis extract + complete culture medium and the other group was primed with 7 µM of CBD-rich cannabis extract + complete culture medium. EqAT-MSCs cultured in a complete culture medium were used as the control. After 24 h of priming, the morphological evaluation and gene expression analysis of cytokines such as interleukin 1 beta (IL-1β), interleukin 6 (IL-6), interleukin 10 (IL-10), interferon-gamma (IFN-γ), and tumor necrosis factor-alpha (TNF-α) were performed. Cell metabolic activity was assessed using the MTT assay, and cellular senescence was evaluated through β-galactosidase activity. The apoptosis assay was carried out using annexin and 7-AAD. In addition to the evaluation of cannabinoid receptors 1 (CB1) and 2 (CB2) gene expression in naïve EqAT-MSCs (P2–P5). Created in BioRender. (https://BioRender.com/q24g045 (accessed on 23 April 2025)).

Eq-ATMSCs (P3) were divided into three groups: the control group (complete culture medium), the group primed with 5 µM of CBD-rich cannabis extract + complete culture medium, and the group primed with 7 µM of CBD-rich cannabis extract + complete culture medium. After 24 h of priming, morphological evaluation and gene expression analysis of cytokines such as interleukin 1 beta (IL-1β), interleukin 6 (IL-6), interleukin 10 (IL-10), interferon-gamma (IFN-γ), and tumor necrosis factor-alpha (TNF-α) were measured on five samples using RT-qPCR. MTT assay, β-galactosidase activity, and apoptosis assay were conducted on three samples (Figure 6). Additionally, the expression of cannabinoid receptors 1 (CB1) and 2 (CB2) was evaluated in the naïve EqAT-MSCs (P2–P5) using four samples per passage.

### 4.2. EqAT-MSCs

The EqAT-MSCs (P2–P5) from healthy horses were obtained from the MSCs bank of the Center for Translational Research in Regenerative Medicine-School of Veterinary Medicine and Animal Science-São Paulo State University. These MSCs were previously characterized, demonstrating the potential for osteogenic, adipogenic, and chondrogenic differentiation, as well as expressing the surface markers CD44, CD90, and CD105, and not expressing CD34 and MHC-II [50].

### 4.3. CBD-Rich Cannabis Extract

The full-spectrum *Cannabis sativa* extract, predominantly rich in CBD, used in this study was supplied by the Maria Flor Cannabis Association (Marília, SP, Brazil). The purity of the CBD-rich cannabis extract was analyzed using High-Performance Liquid Chromatography (HPLC) by DALL Soluções Analíticas (Curitiba, PR, Brazil), revealing 28.12% CBD and 0.80% THC. The extract was diluted in dimethyl sulfoxide (DMSO) at a 1:1 ratio, filtered, and subsequently diluted in DMEM to achieve concentrations of 5 µM and 7 µM, which were utilized in this study.

### 4.4. EqAT-MSCs Primed with CBD-Rich Cannabis Extract

The EqAT-MSCs were thawed and seeded at a density of 5 × 10^4^ cells per well in 24-well plates (Kasvi, Pinhais, PR, Brazil), in duplicate for gene expression analysis, using 0.5 mL of complete medium containing 90% DMEM/F12, 10% FBS (both from Nova Biotecnologia, Cotia, SP, Brazil), 1% penicillin/streptomycin, and 0.5% amphotericin B (both from Gibco, Grand Island, NY, USA). The MSCs were incubated at 37.5 °C in a humidified atmosphere containing 95% air and 5% CO_2_. After 24 h of culture, the supernatant was discarded and the culture medium was replaced for each experimental group: the control group (complete culture medium), the group primed with 5 µM of CBD-rich cannabis extract + complete culture medium, and the group primed with 7 µM of CBD-rich cannabis extract + complete culture medium. The priming was carried out for 24 h. For the MTT assay and β-galactosidase activity analysis, the equine MSCs were seeded into 96-well plates (Kasvi, Pinhais, PR, Brazil) at a density of 1 × 10^4^ cells per well in 100 µL of complete medium and incubated at 37.5 °C in a humidified atmosphere containing 5% CO_2_ and 95% air. After cell adherence, the medium was replaced, and the EqAT-MSCs were primed with 5 µM and 7 µM concentrations of a CBD-rich cannabis extract for 24 h. For apoptosis assessment, the EqAT-MSCs were seeded at the same density in 25 cm^2^ culture flasks (Kasvi, São Pinhais, PR, Brazil) with 5 mL of complete medium. The cells were cultured under the same conditions mentioned earlier until reaching 70–80% confluence. Afterward, the culture medium was replaced, and the EqAT-MSCs were primed with 5 µM and 7 µM concentrations of CBD-rich cannabis extract for 24 h.

### 4.5. Morphological Evaluation

The morphology of the EqAT-MSCs was analyzed using inverted microscopy (LEICA DMIRB, Wetzlar, Germany). Photomicrographs of the unprimed cells (control group) and EqAT-MSCs primed with CBD-rich cannabis extract at concentrations of 5 µM and 7 µM were captured after 24 h of priming.

### 4.6. MTT Assay

To evaluate whether different concentrations (5 and 7 μM) of CBD-rich cannabis extract influence the metabolic activity of EqAT-MSCs, the MTT assay [3-(4,5-dimethylthiazol-2-yl)-2,5-diphenyltetrazolium bromide] (Thermo Fisher Scientific, Waltham, MA, USA) was performed. For this, the EqAT-MSCs were primed with CBD-rich cannabis extract at concentrations of 5 or 7 μM for 24 h. After this period, the culture medium was removed and replaced with the MTT solution, followed by a 4 h incubation under the same conditions. The reagent was then discarded, and 200 µL of DMSO (Synth, Diadema, SP, Brazil) was added to each well to solubilize the formazan crystals. Absorbance was measured at 570 nm using a microplate reader (Biochrom Asys Expert Plus; Biochrom Ltd., Harvard Bioscience, Holliston, MA, USA).

### 4.7. Senescence-Associated β-Galactosidase Activity Assay

To assess β-galactosidase activity in EqAT-MSCs, the Mammalian β-Galactosidase Assay Kit (Thermo Fisher Scientific, Waltham, MA, USA) was used according to the manufacturer’s instructions. EqAT-MSCs were primed with CBD-rich cannabis extract at concentrations of 5 or 7 μM for 24 h. Following priming, the culture medium was removed, and the cells were washed once with phosphate-buffered saline (PBS) (Nova Biotecnologia, Cotia, SP, Brazil). Then, 100 µL of β-galactosidase assay reagent was added to each well. The plates were incubated for 30 min at 37 °C, and absorbance was measured at 405 nm using a microplate reader.

### 4.8. Apoptosis Assay

The apoptosis assay was performed to assess whether priming with CBD influenced the viability of EqAT-MSCs. The evaluation of the apoptosis rate was conducted using the Annexin V-FITC apoptosis detection kit (Invitrogen^TM^, Thermo Fisher Scientific, Waltham, MA, USA) and 7-AAD (BD, Biosciences Pharmingen, San Diego, CA, USA), following the manufacturer’s instructions. In brief, the EqAT-MSCs were primed for 24 h with CBD-rich cannabis extract at concentrations of 5 and 7 μM. After this period, the cells were resuspended in 200 μL of 1× binding buffer and incubated with 5 μL of Annexin V-FITC for 10 min at room temperature in the dark. Subsequently, a wash with 200 μL of 1× binding buffer was performed, and the cells were resuspended in 400 μL of 1× binding buffer. Finally, 10 μL of 7-AAD was added, and the analysis was carried out on a FACS Calibur^TM^ flow cytometer (BD Becton Dickinson and Company, Franklin Lakes, NJ, USA), acquiring at least 10,000 events.

### 4.9. Gene Expression

The gene expression of cytokines *IL-1β*, *IL-6*, *IL-10*, *IFN-γ*, and *TNF-α* were assessed in the control group (P3) and in the EqAT-MSCs (P3) primed with CBD-rich cannabis extract at concentrations of 5 µM and 7 µM using RT-qPCR. Additionally, the gene expression of *CB1* and *CB2* was evaluated in the control group of EqAT-MSCs across different passages (P2–P5).

After 24 h of priming, cell lysis was performed using 1 mL of Trizol^TM^ (Invitrogen^TM^, Thermo Fisher Scientific, Waltham, MA, USA), and the samples were stored at −80 °C for further analyses. RNA extraction with TRIzol reagent followed the manufacturer’s instructions. Subsequently, RNA was eluted using RNA-free water, quantified, and analyzed by spectrophotometry using the Thermo Scientific NanoDrop 2000 equipment (Thermo Fisher Scientific, Wilmington, DE, USA) to determine absorbance ratios at 260/280 nm and 260/230 nm.

For cDNA synthesis, the High-Capacity cDNA Reverse Transcription Kit (Applied Biosystems^TM^, Life Technologies Corporation, Carlsbad, CA, USA) was utilized following the manufacturer’s instructions. The reverse transcription process to obtain the cDNA was conducted using the Veriti 96 Well Thermal Cycler (Applied Biosystems^TM^, Thermo Fisher Scientific, Waltham, MA, USA) employing the following thermocycling conditions: 10 min at 25 °C; 12 min at 37 °C; and 5 min at 85 °C. The resulting cDNA samples were cryopreserved at −80 °C and utilized as templates for PCR reactions.

PCR reactions were performed in duplicate utilizing cDNA generated with PowerUp^TM^ SYBR^TM^ Green Master Mix (Applied Biosystems^TM^, Thermo Fisher Scientific, Waltham, MA, USA)), RNA-free water, and equine-specific primers (Thermo Fisher Scientific, São Paulo, SP, Brazil), designed with Primer Express^TM^ Software v3.0.1 (Applied Biosystems^TM^, Thermo Fisher Scientific, Waltham, MA, USA) (Table 1).

The samples were evaluated using the mean of the following reference genes: beta-actin (*ACTB*), beta-2-microglobulin (*B2M*), glyceraldehyde 3-phosphate dehydrogenase (*GAPDH*), and hypoxanthine-guanine phosphoribosyltransferase (*HPRT*), as described in a previous study [15]. RT-qPCR was performed using the QuantStudio^TM^ 12K Flex Real-Time PCR System (Applied Biosystems^TM^, Thermo Fisher Scientific, Waltham, MA, USA). The relative quantification of the target genes was performed using the ΔΔCt method [51].

### 4.10. Statistical Analysis

The variables of relative quantification, cellular metabolic, and β-Galactosidase activities did not show a normal distribution. Hence, group comparisons were analyzed using the Kruskal–Wallis test (non-parametric), and when significant, medians were compared using Dunn’s test. The variables from the apoptosis were assessed through a one-way analysis of variance (ANOVA) assuming a normal distribution. For all the analyses, group differences were considered statistically significant at *p* < 0.05. All the statistical analyses were conducted using GraphPad Prism version 8, San Diego, CA, USA.

## 5. Conclusions

The naïve EqAT-MSCs expressed CB1 and CB2 receptors. Priming with a CBD-rich cannabis extract demonstrated immunomodulatory effects, with 5 μM CBD upregulating IL-10, TNF-α, and IFN-γ expression, and 7 μM CBD downregulating IL-1β and IL-6 expression. These findings highlight the potential of CBD-rich extracts to modulate the inflammatory and immunomodulatory profiles of EqAT-MSCs. Further in vitro studies are warranted to investigate the signaling pathways underlying these effects, assess the immunomodulatory response of CBD-primed EqAT-MSCs under inflammatory conditions, perform cytokine profiling, and evaluate their secretory activity. Moreover, in vivo investigations are necessary to determine the therapeutic potential of the conditioned medium derived from primed cells in models of inflammation and tissue regeneration.

## Figures and Tables

**Table 1:** Primer sequences used in RT-qPCR reactions.

Gene	Forward	Reverse
*CB1*	GAGCAAGGACCTGAGACATGCT	TGCGCAGTGCCTTCACAT
*CB2*	AAAGAAGAGGCCCCGAAGTC	CCACTGGGTGATTTTCACATCA
*IL-1β*	GCAGCCATGGCAGCAGTA	ATTGCCGCTGCAGTAAGTCA
*IL-6*	AACAACTCACCTCATCCTTCGAA	CGAACAGCTCTCAGGCTGAAC
*IL-10*	CGGCCCAGACATCAAGGA	TCGGAGGGTCTTCAGCTTTTC
*IFN-γ*	CTGTCGCCCAAAGCTAACCT	GGCCTCGAAATGGATTCTGA
*TNF-α*	TTGGATGGGCTGTACCTCATC	GGGCAGCCTTGGCCTTT
*ACTB*	CGGCGGCTCCATTCTG	CTGCTTGCTGATCCACATCTG
*B2M*	CACCCAGCAGAGAATGGAAAG	CGGATGGAACCCAGAGACA
*GAPDH*	GGCAAGTTCCATGGCACAGT	GGGCTTTCCGTTGATGACAA
*HPRT*	GCTCGAGATGTGATGAAGGAGAT	CCCCCTTGAGCACACAGAGT

CB1: cannabis receptor 1; CB2: cannabis receptor 2; IL-1β: interleukin 1 beta; IL-6: interleukin-6; IL-10: interleukin-10; IFN-γ: interferon gamma; TNF-α: tumor necrosis factor-alpha; ACTB: beta-actin; B2M: beta-2-microglobulin; GAPDH: glyceraldehyde 3-phosphate dehydrogenase; HPRT: hypoxanthine-guanine phosphoribosyltransferase.

## Data Availability

The datasets used and/or analyzed during the current study are available from the corresponding author upon reasonable request.
